# An *Anaplasma phagocytophilum* T4SS effector, AteA, is essential for tick infection

**DOI:** 10.1128/mbio.01711-23

**Published:** 2023-09-25

**Authors:** Jason M. Park, Brittany M. Genera, Deirdre Fahy, Kyle T. Swallow, Curtis M. Nelson, Jonathan D. Oliver, Dana K. Shaw, Ulrike G. Munderloh, Kelly A. Brayton

**Affiliations:** 1 Program in Vector-borne Disease, Department of Veterinary Microbiology and Pathology, Washington State University, Pullman, Washington, USA; 2 Department of Entomology, College of Food, Agricultural, and Natural Resource Sciences, University of Minnesota, Saint Paul, Minnesota, USA; 3 Division of Environmental Health Sciences, School of Public Health, University of Minnesota, Minneapolis, Minnesota, USA; University of Nebraska Medical Center, Omaha, Nebraska, USA

**Keywords:** rickettsia, *Anaplasma*, obligate intracellular, effector functions, vector-borne diseases, actin, host-pathogen interactions

## Abstract

Pathogens must adapt to disparate environments in permissive host species, a feat that is especially pronounced for vector-borne microbes, which transition between vertebrate hosts and arthropod vectors to complete their lifecycles. Most knowledge about arthropod-vectored bacterial pathogens centers on their life in the mammalian host, where disease occurs. However, disease outbreaks are driven by the arthropod vectors. Adapting to the arthropod is critical for obligate intracellular rickettsial pathogens, as they depend on eukaryotic cells for survival. To manipulate the intracellular environment, these bacteria use type IV secretion systems (T4SS) to deliver effectors into the host cell. To date, few rickettsial T4SS translocated effectors have been identified and have only been examined in the context of mammalian infection. We identified an effector from the tick-borne rickettsial pathogen *Anaplasma phagocytophilum*, HGE1_02492, as critical for survival in tick cells and acquisition by ticks *in vivo*. Conversely, HGE1_02492 was dispensable during mammalian cell culture and murine infection. We show that HGE1_02492 is translocatable in a T4SS-dependent manner to the host cell cytosol. In eukaryotic cells, the HGE1_02492 localized with cortical actin filaments, which is dependent on multiple sub-domains of the protein. HGE1_02492 is the first arthropod-vector specific T4SS translocated effector identified from a rickettsial pathogen. Moreover, the subcellular target of HGE1_02492 suggests that *A. phagocytophilum* is manipulating actin to enable arthropod colonization. Based on these findings, we propose the name AteA for *Anaplasma* (*phagocytophilum*) tick effector A. Altogether, we show that *A. phagocytophilum* uses distinct strategies to cycle between mammals and arthropods.

**IMPORTANCE:**

Ticks are the number one vector of pathogens for livestock worldwide and for humans in the United States. The biology of tick transmission is an understudied area. Understanding this critical interaction could provide opportunities to affect the course of disease spread. In this study, we examined the zoonotic tick-borne agent *Anaplasma phagocytophilum* and identified a secreted protein, AteA, which is expressed in a tick-specific manner. These secreted proteins, termed effectors, are the first proteins to interact with the host environment. AteA is essential for survival in ticks and appears to interact with cortical actin. Most effector proteins are studied in the context of the mammalian host; however, understanding how this unique set of proteins affects tick transmission is critical to developing interventions.

## INTRODUCTION

Multi-host pathogens often have specific adaptation mechanisms to survive in disparate environments (1). For example, vector-borne microbes must adapt and survive in both vertebrate hosts and arthropod vectors (2). These two environments differ significantly with discrepancies in body temperature, nutrient availability, cell types infected, physiological architecture, and immunological pressures (2). Most of our understanding of tick-borne pathogens focuses on interactions with mammalian hosts, as this is where disease occurs. However, the mammal represents only half of the lifecycle for tick borne pathogens. In contrast, little is known about how tick-vectored pathogens mediate interactions with the arthropod. With over 680 million years of evolution separating ticks from mammals (3), our understanding of mammal-pathogen interactions cannot simply be transposed onto ticks (2).

Adapting to different environments is especially critical for obligate intracellular rickettsial pathogens, which are intimately dependent on both arthropod and vertebrate host cells. One of the most common tick-borne rickettsial human pathogens in the United States is *Anaplasma phagocytophilum*, which causes human granulocytic anaplasmosis (4). To complete its lifecycle, *A. phagocytophilum* must transit between *Ixodes scapularis* ticks and mammalian hosts. Interestingly, *A. phagocytophilum* host cell tropisms are not equivalent between mammals and ticks. In mammals, the bacterium preferentially infects neutrophils. In contrast, *A. phagocytophilum* must infect and traverse the tick midgut, travel through the hemocoel, and infect the salivary glands of the arthropod (5, 6). Given the disparities in the host environment and cell tropisms, it is expected that *A. phagocytophilum* would have unique expression profiles adapted for each situation. Transcriptional studies demonstrated that *A. phagocytophilum* differentially transcribes 41% of its genes when growth in tick cells was compared to mammalian cells (7, 8). Transposon mutagenesis found that many *A. phagocytophilum* genes are dispensable for growth in the human monocyte cell line HL60 (9), and several of these same genes are upregulated during tick cell infection. Altogether, this suggests that the tick-specific genes may be involved in arthropod-pathogen interactions (7, 8).

Rickettsial pathogens primarily manipulate host cell biology through effector proteins that are delivered to the host cytosol with a type 4 secretion system (T4SS) (10, 11). T4SS effector molecules subvert host cell defenses and modulate a wide variety of host cell processes (12 – 19). A common target of such effectors can be the actin cytoskeleton, which forms the structural scaffolding of the host cell (20). When compared to the model intracellular bacterium *Legionella pneumophila* (1), relatively little is known about the effector repertoire from *A. phagocytophilum* and other rickettsial pathogens. Only five *A. phagocytophilum* T4SS translocated proteins have been identified to date, and none in the context of tick colonization (16, 17, 21 – 23). The machine learning algorithm OPT4e predicts that *A. phagocytophilum* encodes 48 candidate effectors (24). Fifteen of these candidate genes show unique expression patterns during mammalian and tick cell infection (7, 8). Putative effector HGE1_02492 (APH_0546 in *A. phagocytophilum* strain HZ) demonstrated the highest transcriptional increase when grown in tick cell culture, relative to mammalian cells. Herein, we show that HGE1_02492 is critical for growth in tick cells and colonization of ticks *in vivo*. Further, HGE1_02492 is deliverable by the T4SS into host cell cytosol, where it associates with cortical actin filaments, through multiple sub-domains of the protein. Based on our findings, we propose the name AteA, for *Anaplasma* (*phagocytophilum*) tick effector A, and will henceforth refer to HGE1_02492 as AteA. Altogether, we have identified and characterized the first arthropod-vector specific effector from *A. phagocytophilum*, which targets the eukaryotic cytoskeleton.

## RESULTS

A protein of 1,103 amino acids (~120 kDa) is encoded by *ateA* (HGE1_02492) and appears to be unique to *A. phagocytophilum*. It has 100% sequence identity with APH_0546 from a different strain of *A. phagocytophilum,* strain HZ. *In silico* analysis of AteA predicts that most of the protein is highly disordered, with an N-terminal globular domain (25) and there are two regions containing tandem repeats starting at amino acids 431 and 702 (26).

### Expression of ateA is specific to growth in tick cells

**Fig 1:**
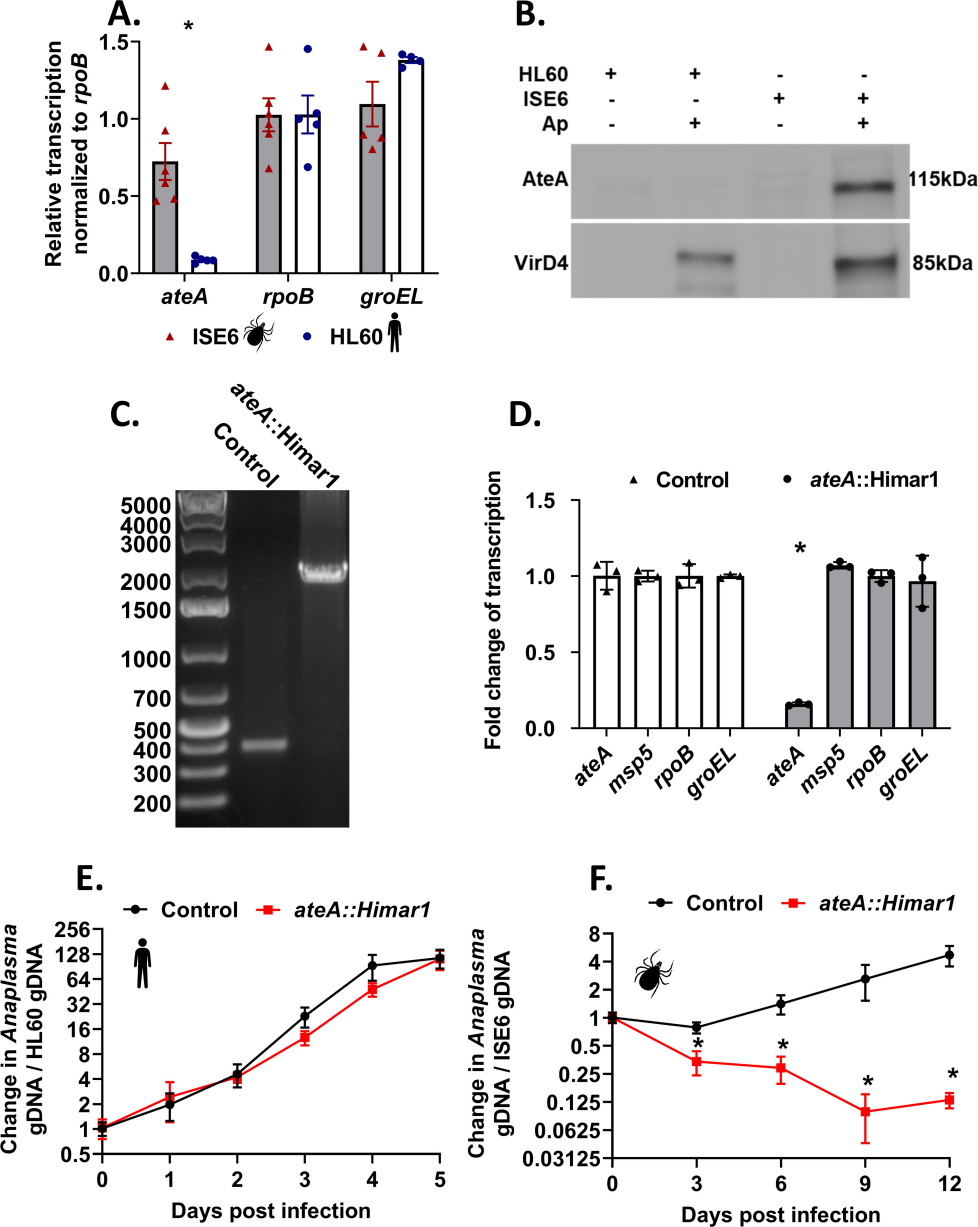
*ateA* is essential for *A. phagocytophilum* survival in tick cells, but dispensable within human cells. (**A**) *A. phagocytophilum* gene expression during growth within tick ISE6 and human HL60 cells. Transcription of *ateA* and housekeeping genes *rpoB* and *groEL*. (**B**) α-AteA (top) and α-VirD4 (bottom) western blot analysis of mock-infected and peak wild-type *A. phagocytophilum* infected human HL60 and tick ISE6 cells. Lanes were equally loaded with 1 × 10^5^ host cells. (**C**) Agarose gel electrophoresis of PCR amplicons flanking the transposon insertion site in the *ateA* gene. (**D**) Transcriptional analysis from *A. phagocytophilum* transposon mutants during culture with tick ISE6 cells. Mutants contain the transposon inserted in a neutral intergenic location (control), or within the *ateA* gene. Transcription measured by qRT-PCR of *ateA, msp5, rpoB,* and *groEL*. Transcription normalized to *rpoB*. Results shown are the mean of three biological replicates with two technical replicates each ±SD. *P < 0.05 (Student’s *t*-test). (**E and F**) Growth of *A. phagocytophilum ateA* or control strain in panel E human HL60 cells and (F) tick ISE6 cells. Bacterial burden was measured as *Anaplasma* gDNA vs host cell gDNA via qPCR. Data shown are the mean of three biological replicates with two technical replicates each ±SD and is representative of three experimental replicates. *P < 0.05 (Mann-Whitney *t*-test).

Previous transcriptomics studies using tiling arrays demonstrated that *ateA* was upregulated during *A. phagocytophilum* culture in ISE6 tick cells and was minimally expressed during growth in the human monocyte-like cell line HL60 (7, 8). Repetitive sequences, like those found in *ateA*, can artifactually inflate transcriptional signals in tiling arrays. We, therefore, used qRT-PCR with primers targeting non-repetitive sequences to quantify *ateA* transcription patterns from *A. phagocytophilum* grown in HL60 and ISE6 cells. Housekeeping *A. phagocytophilum* genes, *rpoB* and *groEL*, are equally expressed when grown in either mammalian or tick cell lines (7, 27), and were, therefore, used as baseline controls for expression. Expression of *ateA* was >8-fold higher during growth in tick cells than in mammalian cells as measured by qPCR (Fig. 1A). To examine AteA protein production, mock-infected and peak wild-type *A. phagocytophilum* infected human HL60 and tick ISE6 cells were examined by western blot for AteA and the T4SS protein VirD4. While VirD4 was detectable during both mammalian and tick cell infection, the AteA protein was only detected from the infected ISE6 lysates (Fig. 1B). Our findings demonstrate that AteA is specifically produced during tick cell infection.

### *A. phagocytophilum* survival in tick cells is dependent on ateA expression

The tick-specific expression pattern of *ateA* led us to ask if it impacts growth in tick cells. For this experiment, we used several tools available to us, including an *ateA* insertion mutant that we previously isolated from a *A. phagocytophilum* Himar1 transposon mutant library (9). As a control strain, we used another mutant, which contains the Himar1 transposon in an intergenic location. This strain has been shown to be phenotypically equivalent to wild-type *A. phagocytophilum* (27, 28). The purity of the *ateA*::Himar1 mutant was examined by PCR bridging the insertion site. The product amplified from *ateA*::Himar1 DNA lacked amplicons of the expected size for the wild type (~400 bp) and contained only the larger (>2,000 bp) product reflecting the Himar1 transposon insertion in *ateA* (Fig. 1C). Our analysis confirmed that transcription of *ateA* was significantly decreased in the *ateA*::Himar1 mutant, but transcription of housekeeping genes, *rpoB* and *groEL,* and the conserved *Anaplasma* gene encoding major surface protein 5 (*msp5*) were all unaffected (Fig. 1B). To test whether the *ateA*::Himar1 mutation impacted growth in the ISE6 cell line, both HL60 and ISE6 tick cells were infected with the *ateA*::Himar1 mutant and growth rates were compared to the intergenic transposon control strain. We found that *ateA*::Himar1 growth in HL60 cells was comparable to the control strain (Fig. 1E), but *ateA*::Himar1 growth in ISE6 cells was significantly attenuated, indicating *ateA* is necessary for survival in tick cells (Fig. 1F).

### Expression of ateA is necessary for *in vivo* tick colonization

**Fig 2:**
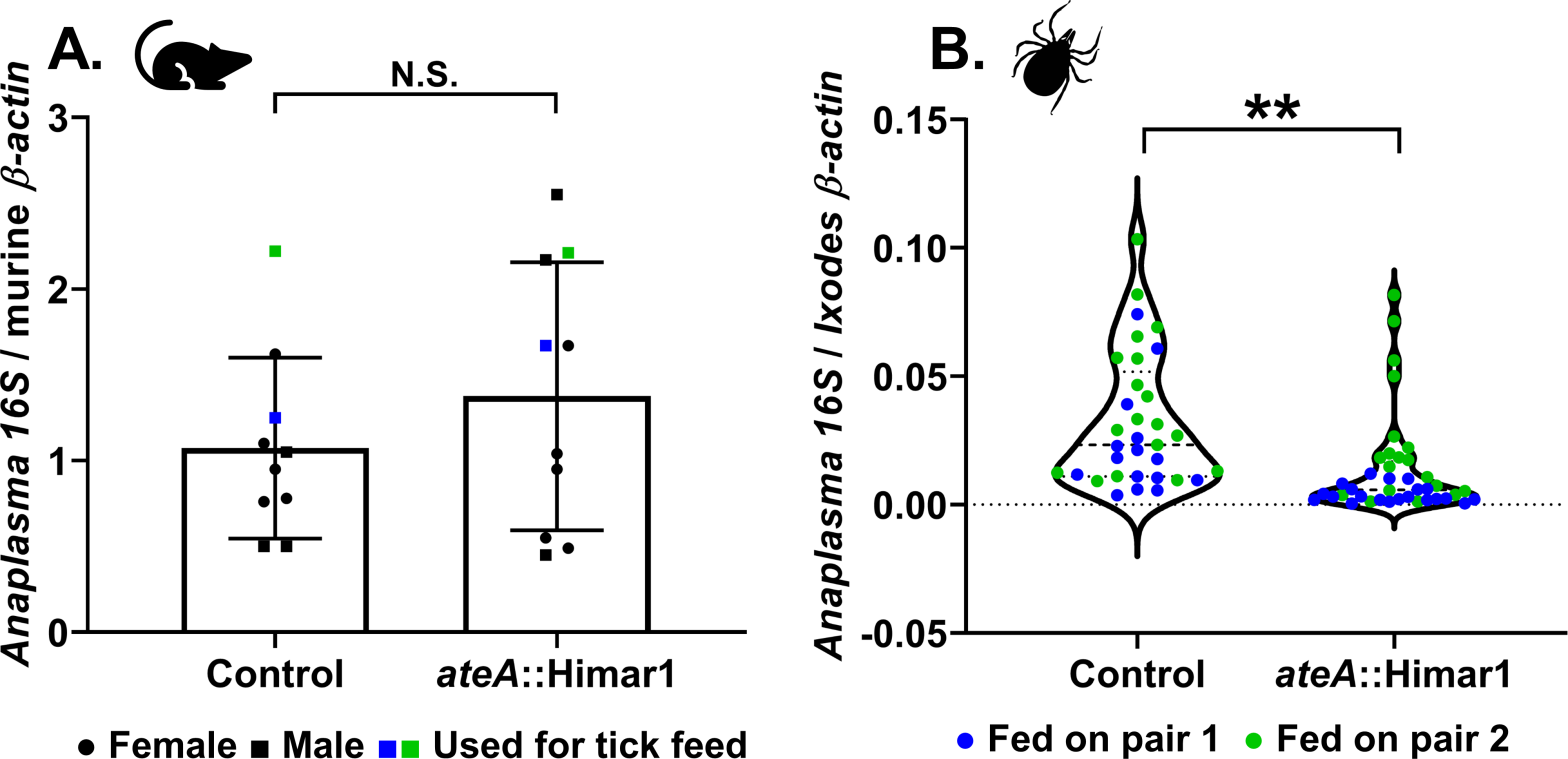
*ateA* is dispensable for murine infection, but mutation attenuates tick acquisition. (**A**) *Anaplasma* burden in mouse blood 7 days post infection by intraperitoneal inoculation with 1 × 10^8^ host cell free *A. phagocytophilum ateA*::Himar1 or control. Blood samples were processed for DNA isolation and bacterial burden was measured by qPCR of *A. phagocytophilum* 16S rDNA relative to mouse actin by ΔΔCt. Each strain was tested in five male (squares) and five female (circles) mice and samples were tested in duplicate. Mice used for tick feeding are indicated by blue and green symbols. Blue symbols indicate experimental replicate 1. Green symbols indicate experimental replicate 2. (**B**) *Ixodes scapularis* larvae were infected by feeding to repletion on *A. phagocytophilum* burden-matched infected mice. Whole replete *I. scapularis* larvae were processed for RNA. Bacterial loads were measured by *A. phagocytophilum* 16S rRNA levels relative to mouse actin via qRT-PCR. Data represent ticks from two burden matched mouse pairs indicated in blue and green for two experimental replicates. Blue symbols indicate experimental replicate 1. Green symbols indicate experimental replicate 2. From each mouse, 17–20 individual ticks were collected as biological replicates, and each qRT-PCR was performed in duplicate. **P < 0.005 (Welch’s *t*-test).

To examine the importance of *ateA in vivo,* we infected mice with either the control (intergenic transposon strain) or the *ateA*::Himar1 mutant strain. No colonization defect was observed in mice, indicating that *ateA* is dispensable during mammalian infection (Fig. 2A). In contrast, larval *I. scapularis* ticks that fed to repletion on *A. phagocytophilum* burden-matched mice acquired significantly less of the *ateA*::Himar1 mutant when compared to the control. This indicates that *ateA* is critical for *A. phagocytophilum* colonization of the tick (Fig. 2B).

### AteA *is a T4SS substrate*

**Fig 3:**
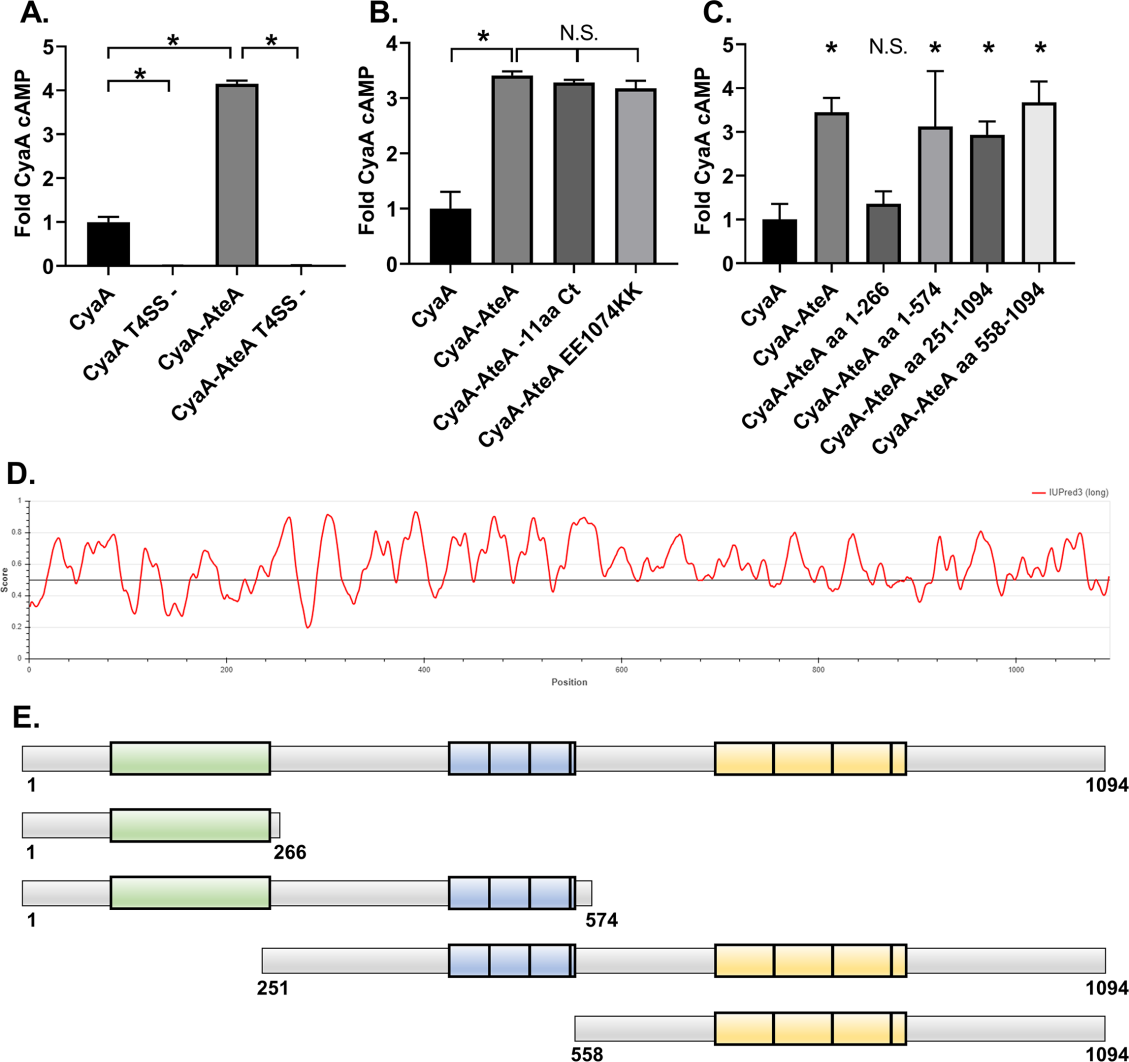
AteA is recognized and secreted by a T4SS. (**A–C**) THP-1 cells were infected with a *L. pneumophila* strain expressing the indicated Cya-fusion proteins for 1 h. cAMP concentrations were quantified from infected cell lysates by ELISA and compared as fold change over CyaA alone. (**A**) CyaA and CyaA-AteA expressed in both wild-type *L. pneumophila* Lp02 or T4SS-deficient Lp03 strain (T4SS−). (**B**) C-terminal mutants of CyaA-AteA were expressed in Lp02 and compared to CyaA alone and CyaA-AteA. (**C**) Truncation constructs of CyaA-AteA diagrammed in panel E were expressed in Lp02 and compared to CyaA alone and CyaA-AteA. (**D**) IUPred3 order/disorder plot of AteA protein. (**E**) Diagram of AteA truncation mutants. Potential globular region highlighted in green, two tandem repeat regions highlighted in blue and yellow. (**A–C**) Error bars represent ±SD of the mean of three biological replicates with two technical replicates each, and graph is representative of two repeated experiments. *P < 0.05 (one-way ANOVA).

Several translocated effector prediction algorithms (OPT4e [24], S4TE [29], and T4EffPred [30]) predict that AteA is a T4SS substrate. To empirically test if AteA is translocatable by a T4SS, we used a well-established (31) surrogate assay in *Legionella pneumophila* (32). In this system, the candidate T4SS substrate is fused to adenylate cyclase (CyaA) and expressed in *L. pneumophila*. Candidate effector translocation is detected by the accumulation of cAMP in host cells during *L. pneumophila* infection. CyaA-AteA led to significantly greater cAMP than the control (CyaA alone) (Fig. 3A). Secretion was not detected from the T4SS deficient *L. pneumophila* strain (*dotA*−)*,* indicating translocation of AteA is T4SS dependent.

A secretion signal common to many T4SS translocated proteins is charged residue at the C-terminus (32, 33). We, therefore, removed 11 C-terminal amino acids from AteA or mutagenized two acidic residues to basic residues in the C-terminus and tested secretion. Neither manipulation strategy affected secretion (Fig. 3B), indicating that a different feature of AteA is being recognized by the T4SS. Intrinsically unstructured regions are another feature common among translocated proteins (34). *In silico* analysis of AteA predicts that most of the protein is highly disordered, with only the N-terminal portion scoring for a globular structure (25) (Fig. 3D). The disordered region has two prominent tandem repeat segments containing either 40 or 59 amino acid repeat units (26) (Fig. 3E). We tested four large truncation fragments of CyaA-AteA for translocation (Fig. 3E). Truncations retaining a large relative amount of the disordered region were secreted in our assay. The truncation that removed the disordered region, leaving only the N-terminal globular region, was not translocated. Altogether, this suggests that AteA contains multiple internal secretion signals, or that the unstructured nature of the protein itself is being recognized by the T4SS for translocation (Fig. 3C and E).

### AteA localizes to the cortical actin cytoskeleton, dependent on multiple domains

**Fig 4:**
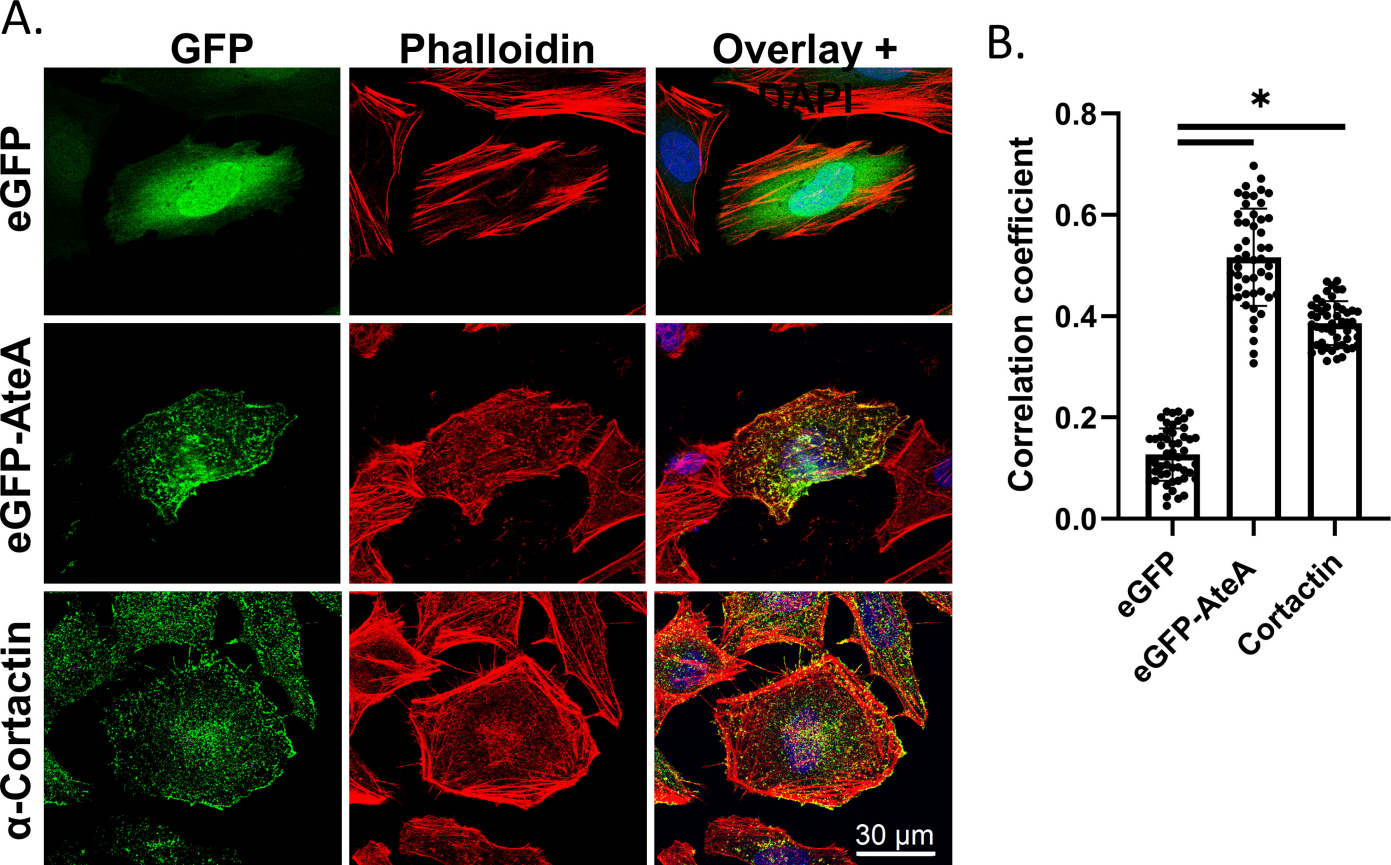
AteA localization resembling cortical actin. (**A**) Confocal images of HeLa cells transiently transfected to express eGFP or eGFP-AteA (rows 1 and 2). Cortical actin binding protein visualized using α-cortactin mouse antibody and Alexa Fluor 488 anti-mouse. Actin was stained with Alexa Fluor 564 phalloidin 36 h post transfection. (**B**) Relative co-localization with phalloidin as measured by Pearson’s correlation (*n* = 20 cells/group). Compared by one-way ANOVA (*P  <  0.05 and ns; not significant).

Since AteA is translocated to the host cell, we examined the eukaryotic host cell structures that AteA may be targeting by ectopically expressing a GFP fusion protein (eGFP-AteA) in HeLa cells. Laser-scanning confocal microscopy revealed that eGFP-AteA appeared as branched filamentous structures, resembling the actin cytoskeleton (Fig. 4). Filamentous actin (F-actin) was visualized using fluorescently labeled phalloidin, which revealed that AteA co-localized with actin filaments (Fig. 4A and B). Many pathogens are known to target actin, which alters host cell processes with the goal of promoting replication and survival. Two prominent F-actin morphologies in cells are cortical actin and stress fibers. Cortical actin resembles a branched web of fibers just under the cell surface. Stress fibers appear as linear actin bundles connecting two anchor points across the cell (35). The highly branched appearance of AteA localization resembles the appearance of cortical actin. For comparison, we stained HeLa cell for the cortical-actin binding protein, cortactin, and phalloidin (Fig. 4A and B). The lack of AteA localization with longer linear actin fibers and the similar appearance to cortactin led us to conclude that AteA preferentially associates with the cortical actin network.

**Fig 5:**
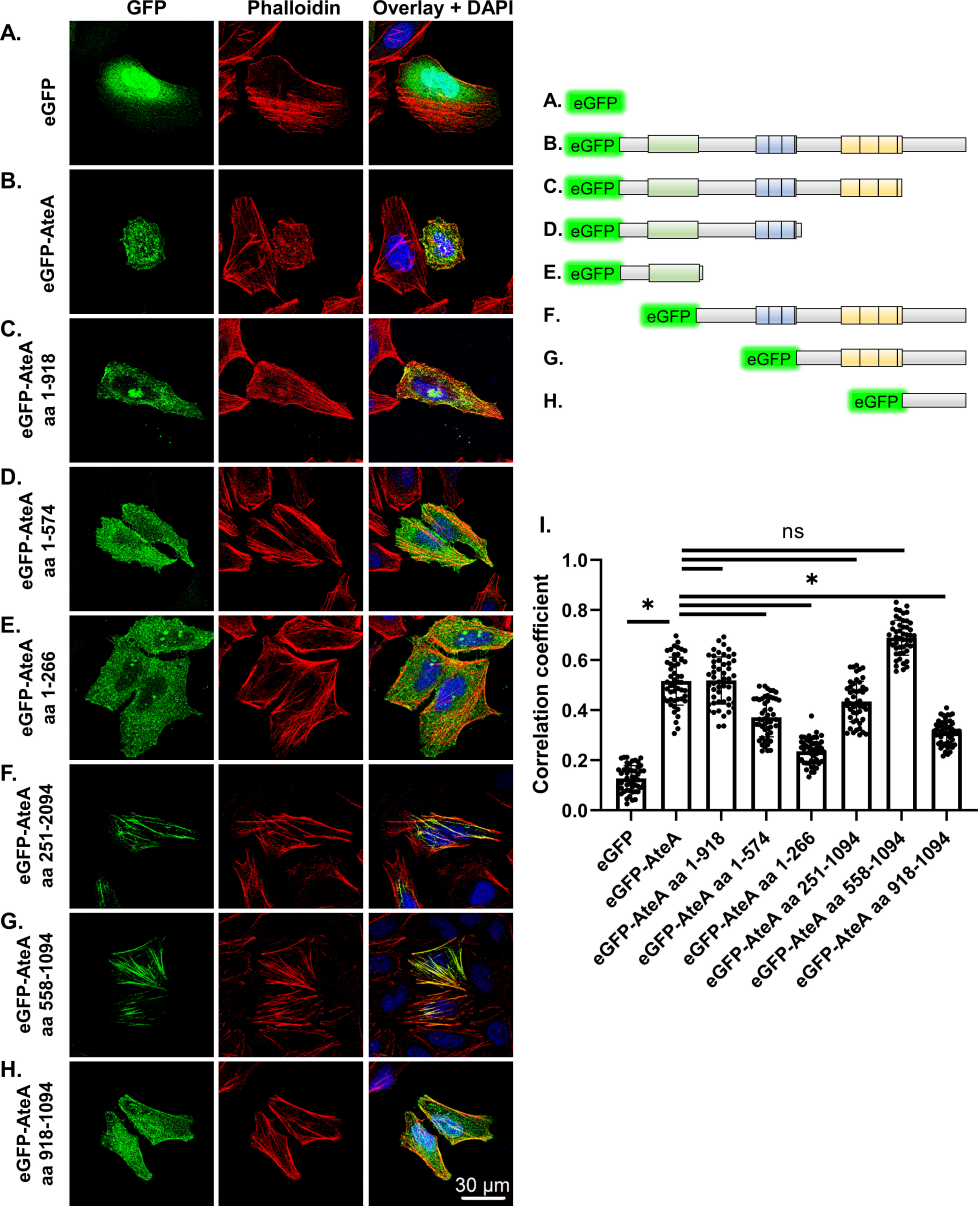
Localization to actin is dependent on the second repeat region, but other regions of AteA influence the pattern. (A–H) Upper right: schematic of eGFP-AteA fusion constructs and truncations used in transfections. (A–H) Left: confocal images of HeLa cells transiently transfected to express eGFP, eGFP-AteA, or an eGFP-AteA truncation construct as diagramed. Cells were stained to visualize actin using Alexa Fluor 564 phalloidin at 36–48 h post transfection. (**I**) Relative co-localization with phalloidin as measured by Pearson’s correlation (*n* = 50 cells/group from three independent experiments). Compared by one-way ANOVA (*P  <  0.05 and ns; not significant).

To identify the regions of AteA responsible for its actin co-localization pattern, truncations of the protein were constructed and ectopically expressed in HeLa cells (Fig. 5). Removal of the C-terminal region following the repeat segments (eGFP-AteA aa1-918) did not change the localization pattern relative to the full-length protein (Fig. 5B and C). Truncations that removed the second tandem repeat region (eGFP-AteA aa 1–574) reduced localization with phalloidin (Fig. 5I) and caused dispersed distribution following the topology of the cell surface (Fig. 5D). The N-terminal globular domain alone (eGFP-AteA aa 1–266) showed a similar localization pattern to eGFP-AteA aa 1–574 (Fig. 5E). These results suggested that the second tandem repeat region (residues 703–918) is necessary for localization with actin. When this region is removed, the protein appears to associate with the cell’s cortex. To test how the other regions of AteA impact the localization, we performed the converse experiment, expressing truncations beginning at the N-terminus. AteA lacking the globular N-terminal region (eGFP-AteA aa 251–1094) lost the appearance of highly branched cortical actin but retained association with actin fibers. This indicates that the N-terminal region of AteA is necessary for the cortical localization (Fig. 5F). Interestingly, the actin fibers associated with AteA lacking the N-terminal globular region did not resemble a cortical actin morphology but appeared more branch-like or distorted than typical actin stress fibers (Fig. 5F). Truncations that removed the central region of AteA containing the first set of tandem repeats (eGFP-AteA aa 558–1094) resulted in the loss of the branched pattern altogether. Instead, the protein localized with long linear actin fibers characteristic of stress fibers (Fig. 5G). The C-terminal portion of AteA alone (eGFP-AteA aa 918–1094) did not localize specifically with phalloidin (Fig. 5H1). To examine the AteA specificity to cortical actin, HeLa cells expressing eGFP-AteA and AteA truncations were stained for the cortical actin-binding-protein cortactin (Fig. S1). Only full length AteA (eGFP-AteA) displayed increased co-localization with cortactin relative to eGFP alone (Fig. S1). However, the incomplete correlation between AteA and cortactin localization (Fig. S1G) suggests that AteA localization to cortical actin may not be specific to the protein cortactin. Taken together, our findings indicate that the second tandem repeat region of AteA is responsible for localization to actin fibers, the central region of the protein alters this actin localization pattern, and the N-terminal domain provides specificity to the cortex. Altogether, these regions function in combination to associate AteA with cortical actin.

## DISCUSSION

Here, we demonstrate that the *A. phagocytophilum* gene encoding *ateA* (i) is highly expressed in the tick environment, (ii) is essential for growth and survival within tick cells, and (iii) is a T4SS translocated substrate that targets the eukaryotic cytoskeleton. Further, *ateA* is necessary for *A. phagocytophilum* acquisition by *I. scapularis* larvae when feeding on an infected host. However, *ateA* was dispensable for growth in mammalian cell culture, and mutation did not affect bacterial burden in mice. To our knowledge, this is the first description of an arthropod specific rickettsial T4SS translocated effector.

AteA joins the few T4SS effectors identified from *A. phagocytophilum* (16, 17, 21 – 23). However, machine learning algorithms predict that *A. phagocytophilum* encodes many more that remain to be tested (24). Among the 48 putative effectors predicted by OPT4e, 15 are differentially transcribed between mammalian and tick cells (7, 8). The three best characterized *A. phagocytophilum* T4SS translocated effectors, Ats-1 (17), AnkA (15, 16), and HGE14 (23), are all downregulated during growth in tick cells (7, 8), suggesting their contributions may be more important during mammalian infection. Our understanding of how *A. phagocytophilum* mediates interactions within mammalian cells is limited, but even less is understood about how the bacteria navigate tick cell biology. A full mechanistic understanding of how rickettsial pathogens facilitate their vector-borne life cycle will require effector identification and characterization in the context of both mammalian hosts and arthropod vectors.

While genetic tools among rickettsial organisms remain limited (36), the *A. phagocytophilum* transposon mutant library (9) allowed us to isolate and test a mutation disrupting *ateA*. Although maintenance of the library in HL60 cell culture precludes mutation of genes essential for mammalian infection, it has equipped us to test the contributions of genes that are important for growth in the tick. This is the second mutant from these libraries shown to have a tick cell-specific phenotype. Mutation of an outer-membrane O-methyltransferase similarly led to a tick cell-specific infection defect (28). Additionally, transposon mutation of a paralogous T4SS component, *virB6-4,* partially attenuated growth in both tick and mammalian cells demonstrating this mutant collection also retains some utility for investigating incomplete phenotypes in mammalian cell models (27). We took the *ateA*::Himar1 mutant beyond cell culture experiments and demonstrated an *in vivo* phenotype through both murine and tick infections that *ateA* is important for bacterial acquisition by ticks from a blood meal. Our work with *ateA*::Himar1 represents the first *A. phagocytophilum* mutant examined in live ticks.

Due to the difficulties in generating recombinant expression systems in an obligate intracellular bacterium (36), efficient T4SS translocation assays using rickettsial organisms have not yet been developed. However, surrogate systems in *Legionella* (31)*, Coxiella* (23)*,* and *Escherichia* (17, 37) have been used to identify rickettsial T4SS substrates. We demonstrated that *L. pneumophila* recognizes and translocates AteA into the host cell cytosol in a T4SS dependent manner. Motifs at the C-terminus often serve as translocation signals for both the *Legionella* and rickettsial T4SS, but they are not universally required and alternative signals can be used (32, 33). AteA contains multiple charged residues in the C-terminus that we determined are dispensable for translocation. Instead, the large, disordered region of AteA was sufficient for translocation. This suggests that the T4SS is recognizing the unstructured nature of the protein or unidentified internal secretion signals. Indeed, disordered regions are a common characteristic among bacterial effectors (34). While the specificity of the *Legionella* T4SS translocation assay cannot be directly projected onto the *A. phagocytophilum* T4SS, *A. phagocytophilum* effectors Ats1, AnkA, and HGE14 also contain intrinsically disordered regions suggesting that this may be a common feature recognized for T4SS translocation (25).

Intracellular bacteria exist among the scaffold of the host cell’s cytoskeleton composed of actin, tubulin, and various intermediate filaments. Pathogens manipulate this cytoskeleton network to promote internalization, evade destruction, alter intracellular trafficking, and disseminate within and between cells (20). Our understanding of how *A. phagocytophilum* interfaces with this cellular scaffold is limited, but differences exist between mammalian and tick cell infection (38, 39). During mammalian infection, the *A. phagocytophilum* vacuole protein AptA recruits intermediate filaments to the *Anaplasma* vacuole. However, transcription of *aptA* is not detectable during *A. phagocytophilum* infection in ticks (39). Actin polymerization is necessary for *A. phagocytophilum* entry into mammalian cells, but during tick cell infection actin is phosphorylated and depolymerized, which is not seen during mammalian infection (38). Although ectopic expression of AteA did not appear to lead to actin depolymerization, this possibility will require further study. We found that AteA localized with branched actin at the cell cortex and was dependent on the predicted tandem repeats and the N-terminal globular domain (Fig. 5). Many other intracellular pathogens are known to manipulate cortical actin to attach to the host cell, induce internalization of the bacteria (20, 40), alter endosome maturation (41, 42), block degradation by the lysosome (43), support the pathogen containing vacuole (44), induce extrusion from the host cell (45), and mediate cell-to-cell bacterial transfer (20, 46). Understanding how AteA manipulates the cytoskeleton to further the *A. phagocytophilum* infection cycle will require further mechanistic dissection.

We demonstrated that *A. phagocytophilum ateA* is specifically important in the tick environment. While examination of AteA localization in tick cells is desired, ectopic expression in ISE6 cells is difficult as transfection efficiency is extremely low, and we were unable to visualize AteA in tick cells. Further, we were unable to visualize AteA during tick infection by immunofluorescence due to high background binding of the α-AteA antibody to the tick cells (not shown). However, we were able to visualize AteA localization with cortical actin in mammalian cells, which may have been possible because actin is one of the most conserved proteins across all eukaryotes (47). Further, mammalian systems have previously been used to investigate multiple actin-targeting T4SS effectors, despite mammals not being the evolutionarily relevant environment (1, 41, 42, 48, 49). The tick-specific expression of *ateA* is highlighted by its dispensability during mammalian infection and severe attenuation of the knockout during tick infection. Why *A. phagocytophilum* requires *ateA* during tick infection remains unclear, but it may stem from the different cell types infected. In mammals, the bacteria preferentially infect phagocytic neutrophils, while in ticks it infects multiple non-phagocytic cell types. AteA may be required to induce internalization or trafficking within tick cells, which neutrophils perform without manipulation.

In summary, we have identified the first tick-specific translocated effector from *A. phagocytophilum* and have shown that it targets cortical actin. While most research on *A. phagocytophilum* focuses on mammalian infection, it is increasingly clear that the mammalian and tick environments are not equivalent. Given that the arthropod vector is the driver of *A. phagocytophilum* transmission, it is critical to understand how these bacteria survive in the tick. We expect that *A. phagocytophilum* deploys a unique repertoire of effectors to navigate the tick environment, with *ateA* being only the first of many to identify.

## MATERIALS AND METHODS

### Bacterial and eukaryotic cell culture

*Escherichia coli* was grown using solid and liquid lysogeny broth (LB) medium with the addition of kanamycin or zeocin (25 µg mL^−1^) antibiotics for selection as needed. *L. pneumophila* Lp02 and Lp03 (dotA−) strains were cultured using N-(2-acetamido)-2-aminoethanesulfonic acid buffered yeast extract medium and solid charcoal buffered yeast extract agar medium. *L. pneumophila* cultures were supplemented with 0.4  mg mL^−1^ iron(III) nitrate, 0.135  mg mL^−1^ cysteine (50), 0.1  mg mL^−1^ thymidine, and when appropriate 50 µg mL^−1^ kanamycin.

HeLa human cervical epithelial cells (American Type Culture Collection [ATCC]; CCL-2) were maintained in Eagle’s Minimum Essential Medium (Corning; 10-010-CV) with 10% fetal bovine serum (FBS: Atlanta biologicals: S11550) and 1× Glutamax (Gibco; 35050061). HL60 human promyelocytic cells (ATCC; CCL-240) and the THP1 human monocyte cell line (ATCC TIB-202) were maintained in Roswell Park Memorial Institute (RPMI) 1640 medium with 10% FBS and 1× Glutamax. Mammalian cell cultures were maintained in a humidified chamber at 37°C with 5% CO_2_. HL60 density was kept between 5  ×  10^4^ and 1  ×  10^6^ and limited to less than 20 passages to prevent differentiation or phenotypic drift.

*A. phagocytophilum* strain HGE1 and mutant lines were cultured in HL60 cells (9). Insertion mutant *ateA*::Himar1 and control strain were isolated from a previously reported *A. phagocytophilum* Himar1 transposon mutant library (9). The control strain contains the Himar1 transposon in an intergenic location and has been shown to be phenotypically equivalent to wild type (27, 28). Infection status of HL60 cells was assessed by Diff-Quick Romanowsky–Giemsa staining. *A. phagocytophilum* were liberated from HL60 cells by 27-gauge needle syringe lysis to generate host-cell-free organisms. Bacterial numbers were estimated as previously described (51, 52).

Tick cells derived from embryonated eggs of the blacklegged tick, *I. scapularis* (Say), ISE6, were grown in L15C-300 medium with 10% FBS (Sigma; F0926), 10% tryptone phosphate broth (TPB; BD; B260300), and 0.1% lipoprotein cholesterol concentrate (MP Biomedicals; 219147680) (53). Infected ISE6 cell cultures were additionally supplemented with 0.25% NaHCO_3_ and 25 mM HEPES buffer (Sigma). Tick cell cultures were incubated at 34°C and 1% CO_2_ (6).

### *Anaplasma phagocytophilum* growth curves

Growth of *A. phagocytophilum* strains in HL60 and ISE6 cells was evaluated as described previously (27, 28). Briefly, HL60 cells were seeded at 5 × 10^4^ cells per well in 24-well plates. The plate was then infected with 5 × 10^4^ host cell-free *A. phagocytophilum* per well for an MOI of 1. Triplicate wells were harvested at the time of inoculation and 1-, 2-, 3-, 4-, and 5-days post inoculation. ISE6 cells were seeded at 3 × 10^5^ cells per well in 24-well plates and allowed to adhere to the plate overnight. Host cell-free preparations of *A. phagocytophilum* strains were done immediately before inoculation and bacteria were suspended in L15C300 supplemented with 0.25% NaHCO3 and 25 mM HEPES. ISE6 plates were inoculated at 3 × 10^6^ *A. phagocytophilum* per well for an MOI of 10. Twenty-four hours post infection, the tick cell media was exchanged for fresh L15C300 + NaHCO3 +HEPES to remove remaining extracellular bacteria, and three wells were collected for initial timepoint. Triplicate samples were collected at subsequent time points and frozen to be processed for gDNA using a QIAGEN DNeasy blood and tissue kit. Change in bacteria and host cell gDNA copies was assessed by qPCR using iTaq universal SYBR green Supermix (Bio-Rad; 1725125) in duplicate reactions. Bacterial gDNA was measured targeting the single copy *A. phagocytophilum* gene *msp5*. HL60 and ISE6 host cell gDNA was measured targeting genes *tlr9* (toll-like receptor 9) and *crt* (calreticulin), respectively (27) (Table S1).

### Transcriptional analysis of ateA

Triplicate samples were collected during the HL60 and ISE6 *A. phagocytophilum* growth curve experiments. Samples were processed to purify RNA using Direct-zol RNA Microprep Kit (ZymoResearch) according to the product protocols for tissue culture samples. The Verso cDNA Synthesis Kit (ThermoFisher) was used to generate cDNA. Transcripts of *ateA*, *rpoB, groEL,* and *msp5* genes were measured by qPCR using iTaq universal SYBR green Supermix (Bio-Rad; 1725125) according to Bio-Rad specified cycle conditions. Transcription of *ateA* was compared between experimental groups by ΔΔCt using *rpoB* as the housekeeping control gene.

### Antibody generation and western analysis

Rabbit antibodies against AteA(aa1056-1075): Cys-ERQMPHKTKSVHELAKQLEE and VirD4(aa720-740): Cys-EDEFGEDRPTDDNDSSNGRLK were generated and purified using synthetic peptides from Pacific Immunology, Ramona, CA, USA (National Institutes of Health animal welfare assurance No. A41820-01).

Proteins from mock-infected and peak wild-type *A. phagocytophilum* infected human HL60 and tick ISE6 cells were separated using 4–15% MP TGX precast gels (Bio-Rad; 4561083). Lanes were equally loaded with 1 × 10^5^ host cells. Proteins were transferred to nitrocellulose membranes and blocked in 5% bovine serum albumin (BSA) in TBS-T (1× Tris-buffered saline + 0.1% Tween 20). Blocked membranes were incubated overnight at 4°C with either primary α-AteA (1:1,000) or α-VirD4 (1:4,000) diluted in TBS-T with 5% BSA. HRP-linked goat α-rabbit IgG secondary antibody (Cell Signaling; 7074) was applied for 2 h at 4°C. Blots were visualized using Pierce ECL western blotting substrate (ThermoFisher; 32209).

### Animal infection

Two gender matched groups of 10, 6-week-old C57BL/6 mice (The Jackson Laboratory) were intraperitoneally infected with either the *ateA*::Himar1 mutant or the control strain at 1  ×  10^7^ host cell-free *A. phagocytophilum* bacteria per mouse. The control strain contains the Himar1 transposon in an intergenic location and has been shown to be phenotypically equivalent to wild type (27, 28). Seven days post infection, 25–50 µL of blood was collected from the lateral saphenous vein. Levels of *A. phagocytophilum* in the blood was measured by qPCR (16S rRNA relative to mouse *β-actin* [51, 54]) (Table S1). Uninfected *I. scapularis* larval ticks were purchased from Oklahoma State University (Stillwater, OK, USA). Ticks were housed at 23°C with 16/8 h light/dark photoperiods in 95–100% humidity. As sources of tick acquisition for *A. phagocytophilum*, two burden-matched-pairs of mice were selected from the *ateA*::Himar1 and control strain infected mice. Each mouse was individually housed and infested with 200 naive unfed *I. scapularis* larvae. Three to seven days post infestation, replete larvae were collected, individually flash frozen with liquid nitrogen, ground with a pestle, dissolved in TRIzol, and processed to purify total RNA according to Direct-zol RNA Microprep Kit protocol. The Verso cDNA Synthesis Kit (ThermoFisher) was used to generate cDNA. Levels of viable *A. phagocytophilum* in the ticks were measured by quantifying *A. phagocytophilum* 16S rRNA relative to *I. scapularis β-actin* transcripts by qRT-PCR (Table S1) by absolute quantification (51, 54).

### Plasmid construction

Full length and truncations of the *ateA* open reading frame were amplified from *A. phagocytophilum* genomic DNA with Gateway compatible primers (Table S1). Amplicons were introduced into pDONR/Zeo by BP Clonase (Invitrogen). Sequence confirmed inserts were then transferred to destination expression vectors with LR Clonase (Invitrogen). For ectopic expression in mammalian cells, we used a Gateway compatible version of pEGFP-C1 (Clontech) and pEZYegfp (Addgene). To create a Gateway destination vector for use in translocation assays (pJC125DEST), the Gateway attR cassette was inserted into the CyaA translational fusion construct pJC125 (55) at a *SmaI* restriction site. CyaA fusion constructs were introduced to *L. pneumophila* by electroporation.

### Translocation assay

THP-1 cells were seeded at 5 × 10^5^/mL in 24-well plate and differentiated to macrophage-like cells by treatment with 200 nM phorbol 12-myristate 13-acetate (Sigma) for 18 h. Transformed *L. pneumophila* cells were grown overnight to an OD_600_ of 2.0, at which point the bacteria were in post-exponential phase, highly motile, and infectious. Expression of the CyaA fusion proteins was induced by adding 1 mM IPTG for 1 h, and motility was verified by microscopy of wet mounted samples. Cell culture medium was used to dilute *L. pneumophila*, which was then used to infect THP-1 cells at an MOI of 1. One hour post infection, cAMP was extracted and quantified as previously described (56) using the cAMP Parameter Assay Kit (R&D Systems).

### Immunofluorescence

HeLa cells were transfected using FuGENE 6 Transfection Reagent at a 3:1 FuGENE to DNA ratio. Proteins were expressed for 36–48 h, then fixed in 4% paraformaldehyde. Fixed cells were permeabilized with 0.1% Triton X-100 for 15 min and washed three times in phosphate buffered saline (PBS). To visualize actin, cells were incubated with Alexa Fluor 568 phalloidin (ThermoFisher Scientific) in PBS containing 1% BSA for 30 min. To visualize cortical actin, we stained for the cortical actin-binding protein, cortactin, α-cortactin (p80/85) mouse monoclonal antibody 4F11 (MilliporeSigma) followed by Alexa Fluor 488 or 594 anti-mouse (Cell Signalling Technology). After each treatment, cells were washed three times for 5 min with PBS and coverslips were mounted on slides using Vectashield mounting medium with DAPI (4′,6-diamidino-2-phenylindole). Slides were imaged using a Leica SP8 confocal microscope. Co-localization was analyzed per cell selected in the green channel using Fiji ImageJ with Coloc_2 co-localization plug-in. A total of twenty cells were analyzed per condition.

### Statistics

All *in vitro* experiments were performed with three biological replicates, measured in technical duplicate assays, and experiments were repeated three times to ensure reproducibility of findings. *In vivo* experiments used 10 independently inoculated mice per group. From each mouse, 17–20 ticks were collected. Two burden matched mice pairs were used for experimental replicates of the tick feeding. Data were expressed as means and graphed with standard deviation. Data points were analyzed with a Student’s *t*-test (Mann-Whitney) or ANOVA for *in vitro* experiments and *in vivo* experiments were analyzed with an unpaired Welch’s *t*-test. Statistical analysis was performed and graphed with GraphPad Prism version 9.0. A *P*-value of <0.05 was considered statistically significant.

## SUPPLEMENTAL MATERIAL

Figure S1 mbio.01711-23-s0001.tif Localization to cortical actin depends on the N-terminal region of AteA.

Table S1 mbio.01711-23-s0002.pdf Oligonucleotides used in this study.

ASM does not own the copyrights to Supplemental Material that may be linked to, or accessed through, an article. The authors have granted ASM a non-exclusive, world-wide license to publish the Supplemental Material files. Please contact the corresponding author directly for reuse.
