# Supplementary figures and images for: An Anaplasma phagocytophilum T4SS effector, AteA, is essential for tick infection
Source: mBio. 2023 Sep 25;14(5):e01711-23. doi: 10.1128/mbio.01711-23 (PMC10653876; doi:10.1128/mbio.01711-23)

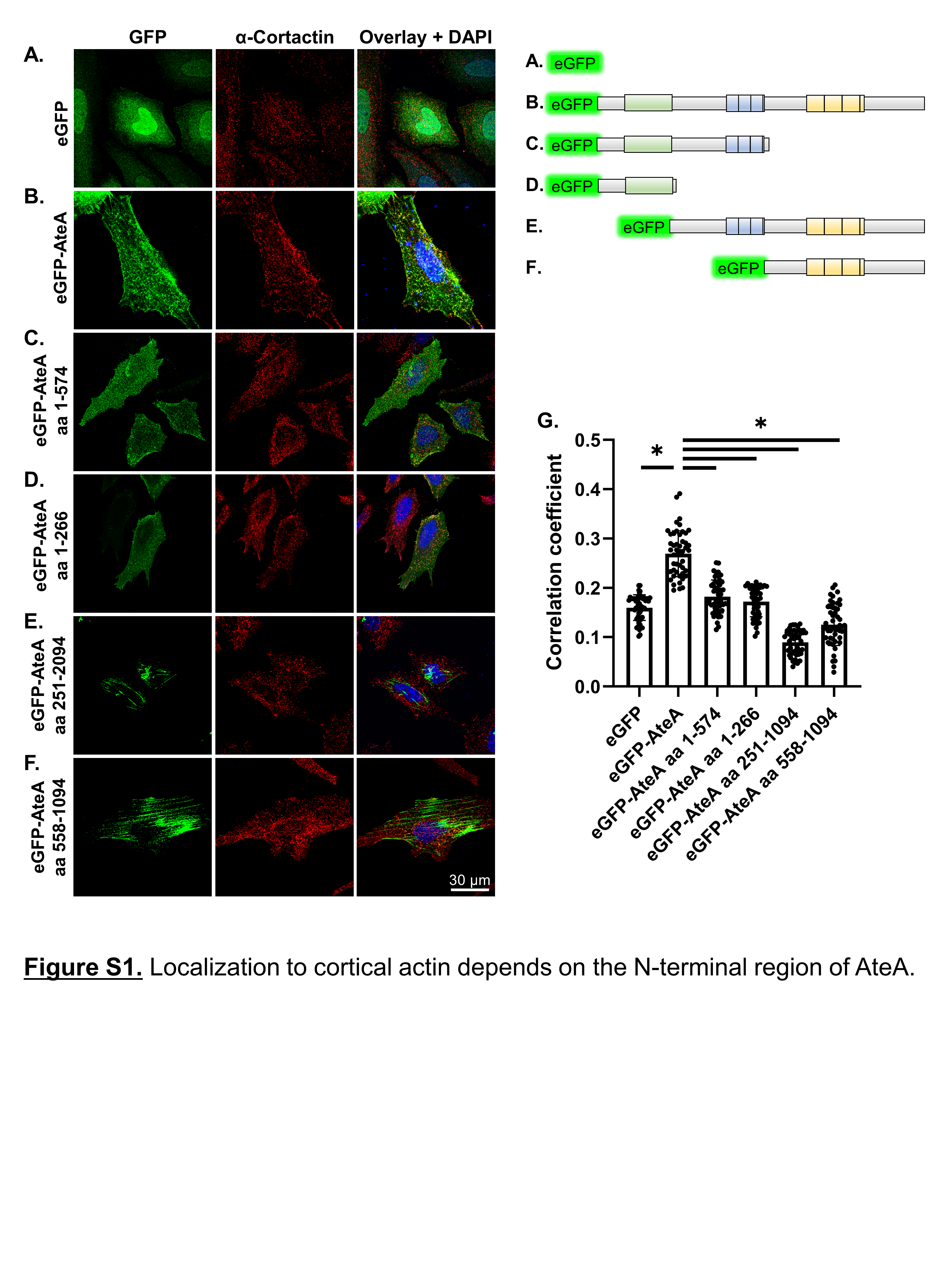

Supplement: Figure S1 — Localization to cortical actin depends on the N-terminal region of AteA. [file mbio.01711-23-s0001.tif]
