# Supplementary material for: An Anaplasma phagocytophilum T4SS effector, AteA, is essential for tick infection
Source: mBio. 2023 Sep 25;14(5):e01711-23. doi: 10.1128/mbio.01711-23 (PMC10653876; doi:10.1128/mbio.01711-23)
Supplement: Table S1 — Oligonucleotides used in this study. [file mbio.01711-23-s0002.pdf]

Supplemental Table 1. Oligonucleotides used in this study

Name	SEQ	Purpose
msp5 RT-qPCR F	TGCGGAACTTGGTATGGTATC	Quantify msp5 transcripts
msp5 RT-qPCR R	CTCATTTAACCTTTCAACAGTGTCA	
groEL F	AGGGAGGTAGTACGCATCCTAGA	Quantify groEL transcripts
groEL R	TGTGATCTCTGGCGACCCATAA	
rpoB F	GGCCTATGGTGCTGCTTATAC	Quantify rpoB transcripts
rpoB R	CCACACTCGAAGTTGCTATCC	
2492 qPCR F	GGCCTTGCTGCTTCCATAACC	qPCR of HGE1_2492 primers
2492 qPCR R	CTCTGTAGAAGGTGTTGCGTCTTC	
msp5 qPCR F	AGATGCTGACTGGGGATGAG	quantification of msp5 Ap DNA
msp5 qPCR R	TCGGCATCAACCAAGTACAA	
crt ISE6 F	GTCAAGTCCGGCACAATCT	quantification of crt gene in ISE6 DNA
crt ISE6 R	CATCTTCTTCTCGGCATCCTT	
tlr9 F	CCCAGTCTTGGACTCAGAATTAG	quantification of tlr9 gene in HL60 DNA
tlr9 R	GGTATAGCCAGGGATTGGTTAAG	
Ap 16S-FL_F	TCCTGGCTCAGAACGAACG	Amplify full Ap 16s for qPCR standard
Ap 16S-FL_R	GTCACTGACCCAACCTTAAATGG	
M.m. B-actin_qPCR-R	ACGCGGGAGGAAGAGGATGCGGCAGTG	Quantify mouse actin transcripts
M.m. B-actin_qPCR-F	ACGCAGAGGGAAATCGTGCGTGAC	
Ap16s_qPCR-F	CCCTAAGGCCTTCCTCACTC	Quantify Anaplasma burden in mouse blood
Ap16s_qPCR-R	CAGCCACACTGGAACTGAGA	
ISE6 actin full R	TACTGTAAAAACAATTTTTATTCCACCAATGAAG	Amplify ISE6 actin for qPCR standard.
ISE6 Actin Full F	ATTTCTTTACCATATTTGGAAGTACGCCACG	
I.s. actin_qPCR-F3	GCCGGGACCTTACAGACTATC	Quantify Ixodes scapularis tick actin primers
I.s. actin_qPCR-R3	CACGGACAATTTCACGCTCG	
Ap16s_qPCR-tick F	AAGCACTCCGCCTGGGGACT	Quantify Anaplasma burdon in ticks
Ap16s_qPCR-tick R	CCATGTCAAGGAGTGGTAAGG	
ChUp & out	ATTATCTTCCTCTCCCTTGCTGACC	PCR and sequencing outward from Himar1 transposon
HGE1_02492 F	GGGGACAAGTTTGTACAAAAAAGCAGGCTTAATGGGAAAATTAACAAAAATC	Gateway compatable HGE1_02492 cloning primers
HGE1_02492 R With Stop	GGGGACCACTTTGTACAAGAAAGCTGGGTACTAGAAACGTGCCCTTGATG	
2492_EE1074KK_F	GAATTAGCAAAACAACTTAAGAAAGGAGGAGTTCTTACGCAAGTGCTTGC	Site directed primers to reverse HGE1_02492 C-terminal charges
2492_EE1074KK_R	GCGTAAGAACTCCTCCTTTCTTAAGTTGTTTTGCTAATTCATGTACAGAT	
2492-11aa C-term GW_R	GGGGACCACTTTGTACAAGAAAGCTGGGTATTAAAGCACTTGCGTAAGAACTCCTCCTTC	Clone HGE1_02492 removing 11aa from the C-teriminus
2492 aa 251-1094 F	GGGGACAAGTTTGTACAAAAAAGCAGGCTTAAGCGCTTCTAAGCACGATGG	Clone HGE1_02492 fragments into pDONR via gateway
2492 aa 558-1094 F	GGGGACAAGTTTGTACAAAAAAGCAGGCTTAAGTATGCTGACAAACAAACCTACTACC	Clone HGE1_02492 fragments into pDONR via gateway
2492 aa 1-266 R	GGGGACCACTTTGTACAAGAAAGCTGGGTACTACTGCTTATTAGAGGAATTAGACTCTTC	Clone HGE1_02492 fragments into pDONR via gateway
2492 aa 1-574 R	GGGGACCACTTTGTACAAGAAAGCTGGGTACTAGTTTTTCAAAGATTTGGGTTCGGG	Clone HGE1_02492 fragments into pDONR via gateway
2492 918 C-term GW F	GGGGACAAGTTTGTACAAAAAAGCAGGCTTAAGTAACAGTGAGATTAAAAGCAAGTCT	Clone HGE1_02492 fragments into pDONR via gateway
2492 aa918 stopCterm GW R	GGGGACCACTTTGTACAAGAAAGCTGGGTACTAAGACTTGCTTTTAATCTCACTGTTACT	Clone HGE1_02492 fragments into pDONR via gateway
2492 5' rev	GCTTCCGCAGAATCATTAGATTGCGGG	sequence from HGE1_02492 into vector
2492 3' F	GTGCAAGCACTACAACAAGAAAGGC	
peGFP C1 seq	CATGGTCCTGCTGGAGTTCGTG	sequencing pEGFP C1
2492 int F	CTCAGGTTCAGTTACGGGTTCTG	Test purity of the HGE1_02492::Himar1 mutant and internal sequencing primers
2492 int R	AGCTCAATAGAAGTAGTAGGCTTGC	
